# Synthesis and Characterization of Store-Operated Calcium Entry Inhibitors Active in the Submicromolar Range

**DOI:** 10.3390/ijms21249777

**Published:** 2020-12-21

**Authors:** Camille Le Guilcher, Tomas Luyten, Jan B. Parys, Mathieu Pucheault, Olivier Dellis

**Affiliations:** 1 Physiopathogénèse et Traitements des Maladies du Foie, Université Paris-Saclay, Rue des Adeles, 91405 Orsay, France; camille.le-guilcher@inserm.fr; 2 INSERM U1193, Rue des Adeles, 91405 Orsay, France; 3 Laboratory for Molecular and Cellular Signaling, Department of Cellular and Molecular Medicine & Leuven Kanker Instituut, B-3000 Leuven, Belgium; tomas.luyten@kuleuven.be (T.L.); jan.parys@kuleuven.be (J.B.P.); 4 Institute of Molecular Science, CNRS, Université de Bordeaux, 33400 Talence, France; mathieu.pucheault@u-bordeaux.fr

**Keywords:** store-operated calcium entry, inhibitor, synthesis, apoptosis

## Abstract

The store-operated calcium entry, better known as SOCE, forms the main Ca^2+^ influx pathway in non-excitable cells, especially in leukocytes, where it is required for cell activation and the immune response. During the past decades, several inhibitors were developed, but they lack specificity or efficacy. From the non-specific SOCE inhibitor 2-aminoethyl diphenylborinate (2-APB), we synthetized 16 new analogues by replacing/modifying the phenyl groups. Among them, our compound P11 showed the best inhibitory capacity with a *K*_i_ ≈ 75 nM. Furthermore, below 1 µM, P11 was devoid of any inhibitory activity on the two other main cellular targets of 2-APB, the IP_3_ receptors, and the SERCA pumps. Interestingly, Jurkat T cells secrete interleukin-2 under phytohemagglutinin stimulation but undergo cell death and stop IL-2 synthesis when stimulated in the presence of increasing P11 concentrations. Thus, P11 could represent the first member of a new and potent family of immunosuppressors.

## 1. Introduction

The control of Ca^2+^ homeostasis is a challenging process for the cell. Indeed, many cellular functions, including contraction, differentiation, and proliferation, are regulated by an increase of the intracellular Ca^2+^ ion concentration [1]. These increases are commonly due to the release of Ca^2+^ ions from internal stores (mainly the endoplasmic reticulum (ER)) and to an influx of Ca^2+^ ions from the extracellular space. The diversity of the Ca^2+^-release and Ca^2+^-influx mechanisms in various cell types is responsible for the variety of Ca^2+^ responses occurring after cell stimulation.

In leukocytes in general, and in lymphocytes in particular, cell stimulation induces the synthesis of inositol 1,4,5-trisphosphate (IP_3_) allowing the release of Ca^2+^ ions out of the ER through the IP_3_ receptor (IP_3_R). This depletion activates a Ca^2+^ influx known as store-operated calcium entry (SOCE) [2]. During the last 15 years, the two main proteins responsible for SOCE have been unveiled: STIM1, the Ca^2+^-sensor protein localized in the ER membrane, and Orai1, the pore-forming protein localized in the plasma membrane. The importance of Orai1 for lymphocyte function appears from the fact that the protein was identified after the discovery of a point mutation creating a non-functional channel, inducing a severe combined immunodeficiency [3]. Thus, even if Orai1 channels are present and the leukocyte repertoire unchanged, the immune system remains deficient.

For more than 30 years, several compounds able to block SOCE have been characterized and developed [4,5,6]. Indeed, a SOCE inhibitor could represent a new way to impair the activation of immune cells and could be of interest in the treatment of chronic inflammatory diseases and leukemias/lymphomas as well as to impair graft rejection or failure.

Among all these molecules, 2-aminoethoxy diphenylborinate (2-APB) has emerged as an interesting compound due to its special properties and the ease to modify its basal structure. Furthermore, 2-APB has intriguing properties: at a concentration between 1 and 5 µM, 2-APB potentiates the leukocyte SOCE but is a full blocker at concentrations above 20-30 µM [7,8]. We previously characterized several 2-APB analogs having either this bimodal function or displaying only inhibiting or only potentiating effects [7,9]. The group of Mikoshiba on the other hand has developed tandem 2-APB molecules with very potent SOCE inhibition capacity, called DPB162-AE and DPB163-AE [6], though DPB163-AE at low concentrations activates SOCE [6] while DPB162-AE also affects ER Ca^2+^-store content in various cell types [10]. Very recently, asymmetrical 2-APB analogues obtained after the exchange of one of the two phenyl groups, or the replacement of the ethanolamine, were tested on MDA-MB-231 cells, which exert an Orai1-dependent SOCE [11]. However, none of these molecules were able to totally block the MDA-MB-231 cells SOCE at 10 µM, and some could induce Ca^2+^ release from internal stores, demonstrating a lack of specificity [11].

Importantly, 2-APB also has a similar biphasic effect on the IP_3_R [12,13] but also inhibits the ER Ca^2+^ pumps (SERCA [14,15]) and can directly open Orai3 channels, allowing an Orai3-dependent SOCE [16,17,18]. These data all underscore that one limit to the use of 2-APB and its analogs is their lack of specificity, implying that any new interesting molecules derived from them must be tested for its effects on other potential targets like IP_3_R and SERCA.

**Figure 1:**
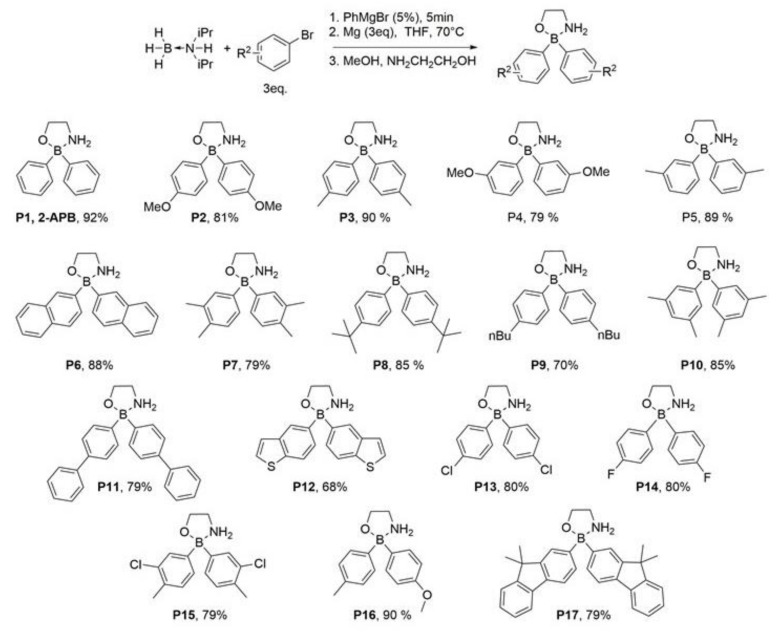
Synthesis of aminoethyl diphenylborinate (APB) analogs using amine borane complex, isolated yields are given after recrystallization.

In this new study, we designed and synthesized 2-APB together with 16 new, non-commercially available 2-APB analogs. In an attempt to get more potent SOCE inhibitors, only the phenyl rings were slightly modified or replaced (Figure 1). Indeed, we previously showed that the presence of the ethanolamine group is associated with the inhibitory capacity of the molecule and that groups larger than phenyl rings impair the potentiation ability [7]. Among these molecules, our interest was particularly drawn to the molecule we called P11 as it is more specific for the SOCE over IP_3_R and SERCA, and inhibits the SOCE with a Ki << 1 µM. This most potent SOCE inhibitor induces apoptosis of phytohemagglutinin (PHA)-stimulated Jurkat cells and impairs the synthesis of interleukin-2, clearly blocking T cell activation. This 2-APB analog P11 could, therefore, represent a new important step in the creation of more potent and specific SOCE inhibitors.

## 2. Results

In our previous work, we used commercially available 2-APB analogs containing various types of modifications of the phenyl groups of the molecule. Replacement of the two phenyl groups by larger groups or the impairment of the free rotation of the phenyl groups by the creation of a carbon bridge between them totally impaired the potentiation ability and gave rise to several interesting, inhibitory molecules [7]. On the contrary, decreasing the size of the two aryl groups only shifted the potentiation/inhibition capacity to higher concentrations [7].

In an attempt to identify and characterize new, stronger SOCE inhibitors, we decided to perform our synthesis of 2-APB analogs by increasing the size of the two aryl groups by adding small groups like methyl or methoxyl groups, or larger ones like other aromatic rings (molecules are depicted in Figure 1).

Using a previously described methodology, these compounds were prepared in a one-pot procedure using air-stable amine borane complexes and Grignard reagents formed under Barbier conditions. The amine borane complexes have been shown to provide easy access to borinic acids and their derivatives. Indeed, by direct addition on the diisopropylamine borane, the diarylaminoborane can rapidly be obtained without undergoing a third addition leading to the triarylborane. In all cases, the diarylaminoborane was readily transformed into the corresponding 2-aminoethylborinate, via simple methanolysis followed by transesterification using 2-aminoethanol.

This approach allowed us to introduce alkyl chain in para (P3, 90%; P8, 85%; P9, 70%) and/or meta position (P5, 89%; P7, 79%, P10, 85%). Other functional groups were perfectly tolerated such as methoxy (P2, 81%; P4, 79%) halides (P13, 80%; P14, 80%, P15, 79%) but also larger (hetero)aromatics (P6, 88%; P11, 79%, P12, 68%, P17, 79%).

**Figure 2:**
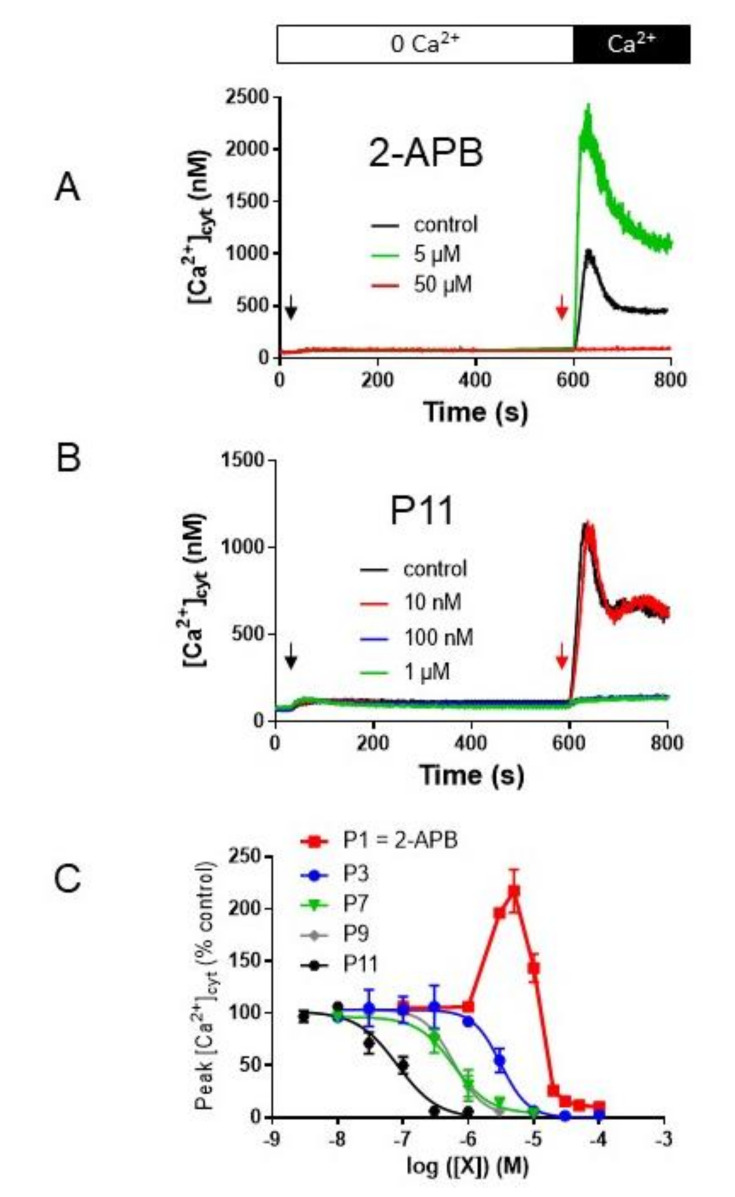
2-APB has a dual effect on Jurkat T cell store-operated calcium entry (SOCE), while the other 2-APB analogs have only an inhibiting effect. (**A**) and (**B**) cytosolic Ca^2+^ concentration ([Ca^2+^]_cyt_) measurement in Jurkat cells using Indo-1 fluorescence. Cells were treated for 10 min with thapsigargin (1 µM, black arrow) to allow Ca^2+^ release from endoplasmic reticulum (ER) and opening of the SOCE channels. After 10 min, 1 mM CaCl_2_ was added, allowing Ca^2+^ entry through the SOCE channels. 2-APB (**A**) or P11 (**B**) at the indicated concentrations was applied 30 s prior to CaCl_2_ (red arrow). Results are representative for five experiments. (**C**) Dose-response curves of compounds P1 (= 2-APB), P3, P7, P9, and P11 on Jurkat cell SOCE. Experiments were performed as in Figure 2A, and the peak [Ca^2+^]_cyt_ was expressed as % of the peak [Ca^2+^]_cyt_ recorded in the absence of any compounds. Compounds were added 30 s prior to CaCl_2_. Results are expressed as mean ± SEM (*n* = 3 to 5 according to the tested molecule).

To visualize SOCE, we performed a classical protocol: Jurkat cells in Ca^2+^-free HBS medium are stimulated by 1 µM thapsigargin (TG, black arrow, Figure 2A) during 600 s to allow the release of Ca^2+^ ions by the ER, and the subsequent opening of the store-operated Ca^2+^ channels (SOCC). Addition of 1 mM CaCl_2_ allowed for a massive entry of Ca^2+^ ions through SOCC leading thus to an increase of the cytosolic calcium concentration ([Ca^2+^]_cyt_). To test 2-APB and its analogues, different concentrations of compounds were added 30 s prior to Ca^2+^ addition.

As shown in Figure 2A, our 2-APB had the typical dual effect on SOCE amplitude: a large potentiation at 5 µM (> 200% of control [Ca^2+^]_cyt_ increase) and an almost total inhibition at 50 µM. No significant differences were found between commercial and our 2-APB (not shown).

Figure 2B depicted the effect of increasing compound P11 concentration: noteworthy the SOCE was totally blocked for concentrations > 100 nM.

We realized the same kind of experiments with all our 2-APB analogues (only four molecules were shown on Figure 2C) and all the molecules have lost the possibility to increase the [Ca^2+^]_cyt_, confirming that enlarging the two phenyl groups impairs the potentiation process, resulting in inhibitors with a *K*_i_ ranging 1000-fold (between 75 nM and 75 µM, Table 1). Noteworthy, we also tested all these molecules on a lymphoblast cell lines devoid of any Orai1 expression due to a double mutations [19] and none was able to induce a [Ca^2+^]_cyt_ increase due to the activation of Orai3 channels as 2-APB can do.

### 2.1. Addition of Methyl and/or Methoxyl Groups

The addition of one methyl on both phenyl rings in para and meta positions respectively for compound P3 (and P5) allowed the creation of inhibitors with an inhibition constant of 2–3 µM (Table 1, Figure 2B). Interestingly, the addition of a second methyl to both phenyl rings significantly increased the inhibition efficacy of the molecule (*p* > 0.05, *n* = 3, compounds P7 (and P10), Figure 2B). Among these two molecules, the compound P10 was the most active with a *K*_i_ ≈ 0.5 µM (Table 1), and complete inhibition was reached at 3 µM.

We also created 2-APB analogs with one methoxy group on the two aromatic rings (compounds P2 and P4). Even if the presence of a methoxy group on the aromatic rings in para position had the same effect as a methyl group (compound P3 ≈ P2), the replacement in meta drastically impaired the inhibitory capacity of the compound P4 (*K*_i_ = 75 ± 21 µM, Table 1) with a maximal inhibition of ≈ 80% (not shown).

Compound P16 represents a chimera between P3 and P2 (Figure 1), bearing one methyl and one methoxy group on either aromatic ring. As compounds P3 and P2 had similar properties, unsurprisingly P16 had a similar *K*_i_ (3.1 ± 0.6 µM, Table 1). 

From these molecules, it seems that the more the two rings are ramified, the more they inhibit the Jurkat cell SOCE.

### 2.2. Fluorine- and Chlorine-Containing Analogs

Compounds P13 and P14 are, respectively, analogs of compound P3 where the methyl groups were replaced by chlorine or fluorine. The presence of a halide only weakly modified the *K*_i_ ≈ 2 µM and 5 µM respectively for compound P13 (Cl) and compound P14 (F) vs. 3.5 µM for compound P3 (Table 1).

Interestingly, the compound P15 which has one methyl + one chlorine on both phenyl rings had a large increase in its inhibitory capacity (*K*_i_ ≈ 0.3 µM, Table 1).

These results confirm that the more ramified is the phenyl group, the more efficient the molecule inhibits the SOCE. However, the use of chlorine or fluorine did not hugely improve the inhibition capacity of the molecules.

### 2.3. Compounds P8 and P9

These two compounds are analogs of P3 with a bulky alkyl residue (*t*Bu, P8) or a linear alkyl chain (*n*Bu, P9). P8 and P9 are better inhibitors with *K*_i_ = 350 ± 40 nM and 641 ± 103 nM, respectively (*n* = 3 for both, Table 1 and Figure 2B). Thus, the addition of larger groups increases the efficacy of the molecules. Noteworthy, compound P7, bearing a 3,4-dimethyl substituent, displayed the same behavior as P9 (Figure 2B).

### 2.4. Fused Ring Analogs

The best inhibitor we previously described was a compound in which the two phenyl groups were replaced by 2 benzothienyl groups attached to the central boron atom by the thienyl parts (*K*_i_ ≈ 0.4 µM, full inhibition at concentration > 3 µM [7]). Our compound P12 is very similar, except that the benzothienyl groups are attached to the boron atom by the benzyl parts. The reversal of this group had no real effect as the *K*_i_ of compound P12 was very similar at 374 ± 96 nM (*n* = 3, Table 1).

The naphtyl analog P6 had a slightly better *K*_i_, 275 ± 17 nM (*n* = 3, Table 1). However, the best inhibitor was obtained when the group was formed by two phenyls bound by a single C-C linker. This compound P11 had a *K*_i_ < 100 nM (75 ± 21 nM, *n* = 3, Table 1) and fully inhibited the SOCE at concentrations below 1 µM (Figure 2B). Interestingly, when we added a gem-dimethyl between the two phenyl groups (compound P17), no effect on the SOCE amplitude was seen for concentrations >3 µM, and higher concentrations disrupted the fluorescence and impaired the measurement. 

Thus, the free rotation of the two phenyl groups on the same side seems important for the high efficacy of compound P11.

### 2.5. P11 Specifically Inhibits Store-Operated Calcium Entry (SOCE) but Does Not Affect the Ca^2+^-Extrusion Mechanisms

As shown in Figure 2A, after TG treatment and addition of extracellular Ca^2+^, the [Ca^2+^]_cyt_ rapidly increased and reached in control conditions a peak of about 1 µM followed by a decay to a new equilibrium around 500 nM. As [Ca^2+^]_cyt_ results from a balance between Ca^2+^ influx and efflux, this decay was due to the activity of the plasma membrane Ca^2+^ ATPases (“PMCA”) and the Na^+^/Ca^2+^ exchanger (“NCX”) counteracting the SOCE (TG has blocked the SERCA activity, impairing Ca^2+^ uptake in the ER). To ensure that compound P11 acted on the SOCE and not on the Ca^2+^-efflux mechanisms, we next performed a Mn^2+^-quenching experiment with Indo-1. Mn^2+^ ions enter the cells through SOCC but cannot be pumped outside the cells by PMCA and NCX; once in the cells, Mn^2+^ bound to Indo-1 and quenched its fluorescence measured at 430 nm. Thus, an increase of the Mn^2+^ influx amplitude was associated with an increase of the Indo-1 quenching speed, and reversely [7,9].

**Figure 3:**
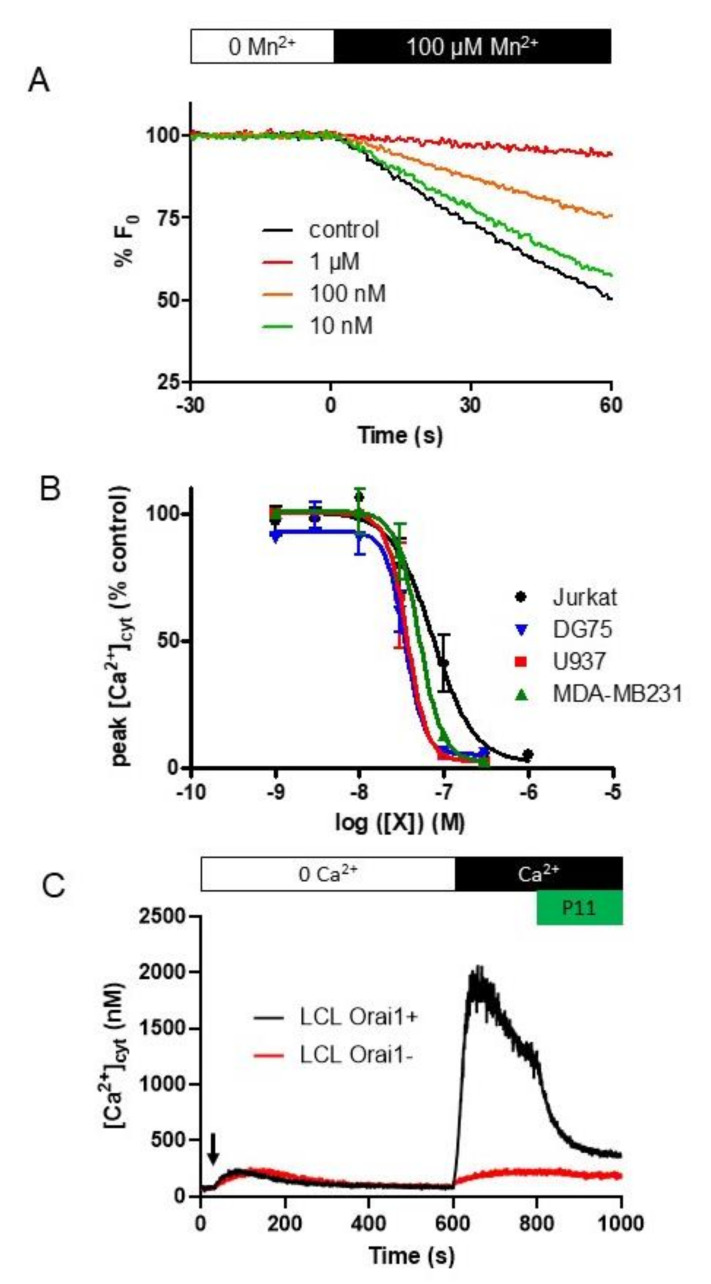
Compound P11 inhibits Mn^2+^ influx in Jurkat cells as well as SOCE in Jurkat cells and other cell types. (**A**) Mn^2+^ quenching of Indo-1 in Jurkat cells is inhibited by increasing P11 concentration. Cells were pre-treated for 10 min with thapsigargin (TG, 1 µM) to allow Ca^2+^ release from the ER and opening of the SOCE channels. Thirty seconds before addition of MnCl_2_, the different [P11] (were added to the bath medium. At t = 0 s, 100 µM MnCl_2_ was added. Fluorescence of Indo-1 was recorded at 430 nm after excitation at 360 nm. Results are representative for three experiments and express the fluorescence relative to that prior to MnCl_2_ addition. (**B**) Dose-response curves for compound P11 on SOCE measured in Jurkat, DG75 (human Burkitt lymphoma cell line), U937 (human monocytic lymphoma cell line), and MDA-MB231 (breast cancer) cells. Experiments were performed for each cell line as in Figure 2A, and the peak [Ca^2+^]_cyt_ was expressed as % of the peak [Ca^2+^]_cyt_ recorded in the absence of any compounds. P11 was added 30 s prior to 1 mM CaCl_2_. Results are expressed as mean ± SEM (*n* = 3 except *n* = 5 for the Jurkat cells) (**C**) Cytosolic Ca^2+^ concentration ([Ca^2+^]_cyt_) measurement in Orai1-expressing lymphoblastoid cell line (“LCL Orai1+”) and Orai1-deficient lymphoblastoid cell line (“LCL Orai1−“) using Indo-1 fluorescence. Cells were treated for 10 min with thapsigargin (1 µM, black arrow) to allow Ca^2+^ release from ER and opening of the SOCE channels. After 10 min, 1 mM CaCl_2_ was added, allowing Ca^2+^ entry through the SOCE channels. 1 µM P11 was added at *t* = 800s. Results are representative for five experiments.

After a 10 min TG treatment to open the SOCC, 100 µM MnCl_2_ was added. Mn^2+^ induced a quenching rate of −0.89 ± 0.05% Fo/s (*n* = 3, Figure 3A). In presence of increasing P11 concentrations added 30 s prior to the Mn^2+^ ions, the Indo-1 quenching rate decreased to reach a ≈ 90% blockade at 1 µM (-0.09 ± 0.01, *n* = 3, Figure 3A). 

From this result, it is clear that the compound P11 targeted the SOCE.

### 2.6. P11 Is Also Able to Block SOCE in Other Cell Types

We also tested P11 on the DG75 (Burkitt B lymphoma), U937 (monocytic lymphoma), and MDA-MB231 (breast carcinoma) cell lines. With the SOCE measured in the same conditions, compound P11 was significantly more efficient on these three cell lines than on Jurkat cells with *K*_i_ = 32 ± 2 nM, 40 ± 5 nM, and 50 ± 5 nM, respectively, for DG75, U937, and MDA-MB231 cells vs. 75 ± 21 nM for Jurkat cells (Figure 3B). Thus, in several cell lines, full inhibition of the SOCE was already obtained at a concentration of 100 nM (Figure 3B).

We next tested P11 efficacy on two lymphoblastoid cell lines (“LCL”) established from an Orai1-deficient with a severe combined immunodeficiency (“LCL Orai1−”) and a healthy (“LCL Orai1+”) patients (Figure 3C [19]). As shown in Figure 3C, in absence of extracellular CaCl_2_, TG induced the Ca^2+^ release from the ER in the two types of cells. However, CaCl_2_ addition after 10 min of TG treatment was only able to induce a massive [Ca^2+^]_cyt_ rise in LCL expressing Orai1 proteins, confirming the SOCE default of the SCID patient. Noteworthy, 1 µM P11 only induced an immediate decrease of [Ca^2+^]_cyt_ in Orai1-expressing LCL and was inefficient in Orai1-deficient LCL. These results confirm that Orai1 is the target of P11 in these cells. 

### 2.7. Selectivity of Compounds P11 and P9

Despite interesting properties, 2-APB is a complex molecule that lacks specificity. Thus, 2-APB was firstly described as an IP_3_R inhibitor [20] but it blocks also SERCA [14] and some TRP channels (TRPC3 for example [21]). Thus, on Jurkat cells, 2-APB has three main targets that play an important role in Ca^2+^ homeostasis. As our molecules are analogs of 2-APB, it was obvious to check whether they also affected SERCA and IP_3_R activity. 

Among our various molecules, we selected to perform experiments on P11 and two structurally close compounds, P8 (Ki ≈ 300 nM) and P9 (Ki ≈ 600 nM). 

Measurements of SERCA-mediated ^45^Ca^2+^ store loading and IP_3_-dependent release from the ER Ca^2+^ store were performed on permeabilized cells in unidirectional conditions. As this approach required the use of cells forming strongly adherent monolayers, we used L15 fibroblasts stably overexpressing IP_3_R1, similarly to our previous work [9]. Unfortunately, P8 induced already a strong Ca^2+^ leak from the ER by an IP_3_-independent pathway, impairing the experiment. 

**Figure 4:**
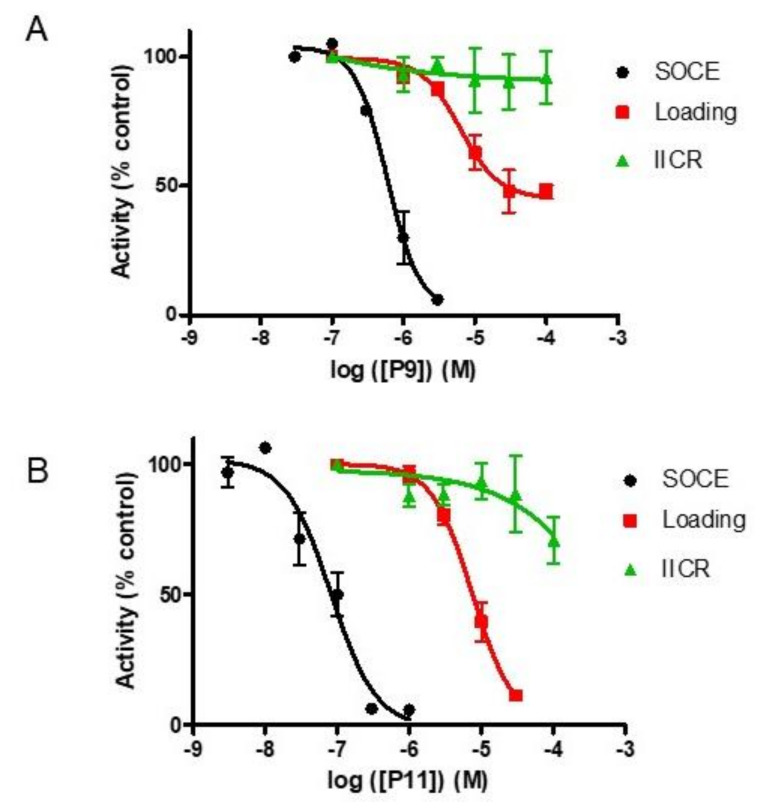
Dose-response curves for compounds P9 (**A**) and P11 (**B**) indicating a preferential inhibition of SOCE over the loading of the ER Ca^2+^ stores by SERCA pumps (“Loading”) and the Ca^2+^ release from the ER by IP_3_Rs (“IICR”). The effects of P9 and P11 on the IP_3_–induced Ca^2+^ release (“IICR”) and the SERCA-mediated Ca^2+^ loading (“loading”) were investigated in permeabilized L15 fibroblasts. The ER Ca^2+^ stores of saponin-permeabilized L15 fibroblasts were loaded until steady-state with ^45^Ca^2+^ and Ca^2+^ release was induced by incubation in a Ca^2+^-free efflux medium in the presence of TG (10 µM). The indicated concentration of P9/P11 were added either during the loading phase for assessing SERCA activity or during the efflux phase before addition of IP_3_ (0.7 µM) to assess IP_3_R activity. Activity was expressed as % of the activity measured in the absence of any compounds. Results are expressed as mean ± SEM (*n* = 3 for “loading, *n* = 5 for “SOCE” and “IICR”).

As shown in Figure 4, both P11 and P9 displayed some inhibitory effects on the activity of SERCA and/or IP_3_R.

Compound P9 did not significantly inhibit IP_3_R activity but was only a partial inhibitor of the SERCA activity: −52 ± 3% (*n* = 4) at 100 µM with a *K*_i_ value of 6 ± 0.3 µM (*n* = 4). Interestingly, below 3 µM, compound P9 thus only inhibited SOCE and quickly lost its efficacy at decreasing concentrations (Figure 4).

In contrast, compound P11 was a weak inhibitor of the IP_3_R (−29 ± 9% at 100 µM, *n* = 3); calculation of the *K*_i_ was however impossible because too high P11 concentrations would be needed to determine this value with sufficient precision. This molecule was also a more complete inhibitor of the SERCA activity, with an almost total inhibition at concentrations > 30 µM (−89 ± 1%, *n* = 3). However, compound P11 was therefore still a hundred times less efficient for inhibiting SERCA activity than for inhibiting SOCE (*K*_i_ = 7.4 ± 2 µM (*n* = 3) vs. 75 ± 21 nM (*n* = 3). Thus, at concentrations < 1 µM, P11 is a specific SOCE inhibitor without any effect on SERCA or IP_3_R in Jurkat cells.

All our subsequent experiments were therefore performed only with compound P11 as it is a specific inhibitor of SOCE that can be used at concentrations <1 µM.

### 2.8. Compound P11 Impairs the Activation of Jurkat Cells

Activation of T cells needs a [Ca^2+^]_cyt_ increase for several minutes to allow the synthesis of interleukin-2 (“IL-2”) and the further activation of the immune functions. Therefore, inhibition of the [Ca^2+^]_cyt_ rise due to SOCE impairs T cell activation and proliferation [7,22].

**Figure 5:**
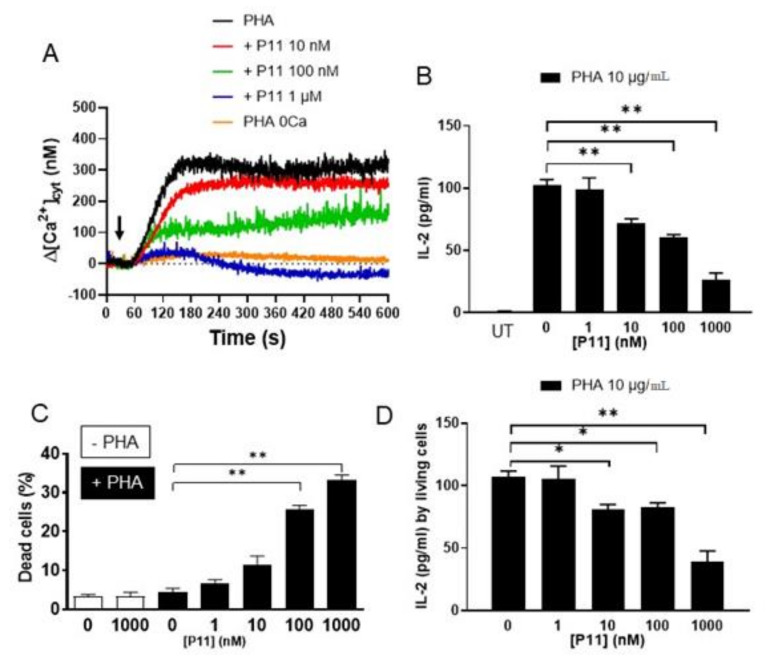
Compound P11 decreases phytohemagglutinin (PHA)-induced Ca^2+^ mobilization and the synthesis of interleukin-2 (IL-2) and increases cell death and decreases by PHA-stimulated Jurkat cells. (**A**) Cytosolic Ca^2+^ concentration ([Ca^2+^]_cyt_) measurement in Jurkat cells using Indo-1 fluorescence. Cells were treated with 10 µg/mL PHA plus increasing [P11] concentrations in presence of 1 mM extracellular CaCl_2_, except “PHA 0Ca” which was obtained by stimulating cells only with PHA in absence of extracellular CaCl_2_. These traces are representative of three repeats. (**B**) Jurkat cells were stimulated by 10 µg/mL PHA in the presence of P11 at the indicated concentrations, UT = untreated cells. IL-2 concentration was measured by ELISA in the cell supernatants. (**C**) Dead cells were estimated after 24 h using trypan blue staining. (**D**) The values of produced IL-2 were corrected for the number of living cells, showing that addition of increasing P11 concentrations at the time of PHA stimulation, progressively decreased the IL-2 synthesis by living cells. Statistically significant differences are indicated by: * *p* < 0.05, ** *p* < 0.01. *n* = 6.

Having shown that compound P11 was able to block the Jurkat cell SOCE, we next performed experiments using phytohemagglutinin (“PHA”). PHA is commonly used to cross-link the T cell receptor allowing the synthesis of IL-2 and thereafter the T cell activation [22]. As shown in Figure 5A, in presence of 1 mM extracellular CaCl_2_, PHA induced a Ca^2+^ mobilization which was largely inhibited by increasing [P11]. Noteworthy, PHA was able to induce a weak Ca^2+^ release from internal stores (orange curve). One micromolar of P11 did not affect this Ca^2+^ release as the PHA-induced Ca^2+^ rise was very similar to the one recorded in absence of extracellular Ca^2+^ ions, showing that P11 only acted on the SOCE and not the ER Ca^2+^ release. However, after reaching a peak at 180 s, the [Ca^2+^]_cyt_ started to decay and decreased to a value beneath the [Ca^2+^]_cyt_ before cell stimulation. Thus, high [P11] concentrations not only blocked the SOCE of PHA-stimulated cells, but also emptied the cytosolic Ca^2+^ content. 

We next studied the effect of increasing [P11] on the neo-synthesized IL-2 concentration in the medium of PHA -stimulated Jurkat cells after 24 h.

In the absence of PHA stimulation, Jurkat cells did not produce any IL-2 (“UT”, Figure 5B). On the contrary, under PHA stimulation, approximately 100 pg/mL IL-2 was detected in the medium (103 ± 4 pg/mL, *n* = 3, Figure 5B) after 24 h. Addition of increasing P11 concentrations at the time of PHA stimulation progressively decreased the IL-2 synthesis to 26 ± 6 pg/mL at 1 µM P11 (−74%, *n* = 3, Figure 5A). By itself, 1 µM P11 was not able to induce IL-2 synthesis (not shown).

As this diminution in IL-2 synthesis can both be explained by the decrease of its synthesis by all cells, or by a decrease in the number of cells that can synthesize IL-2, we next evaluated cell death by trypan blue staining in the presence of PHA stimulation with and without the addition of compound P11.

Under resting culture conditions, the percentage of dead cells measured was lower than 5% (3.4 ± 0.4%, *n* = 5, Figure 5C). After 24h PHA stimulation, the number of dead cells did not significantly increase (4.4 ± 1.0%, *n* = 4). Furthermore, compound P11 by itself was not toxic as no significant increase in dead cells was noticed at a maximal concentration of the compound (at 1 µM: 4.0 ± 0.4%, *n* = 5). 

Addition of increasing concentrations of compound P11 simultaneously with PHA, however strongly increased the proportion of dead cells up to 33.2 ± 1.3% at 1 µM (*n* = 4, *p* < 0.05, Figure 5C). By calculating the amount of IL-2 synthetized by the remaining living cells, it was clear that increasing concentrations of compound P11 decreased this synthesis capacity by ≈ 63% (39.2 ± 8.6 pg/mL vs. 107.6 ± 4.1 pg/mL for PHA-stimulated cells in the absence of P11, Figure 5D). 

These results clearly show that compound P11 has a dual effect on activated Jurkat cell, on the one hand reduction of IL-2 synthesis and on the other an induction of their death. 

### 2.9. Compound P11 Induces Apoptosis of PHA-Stimulated Jurkat Cells

To verify the mechanism of cell death induced by compound P11, we next performed caspase-3 activity assays using the fluorogenic Ac-DEVD-AFC substrate as well as a TUNEL assay.

**Figure 6:**
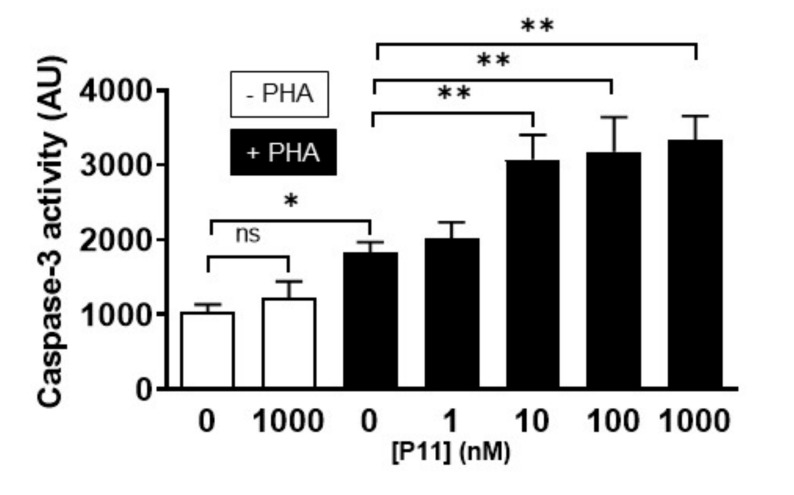
Compound P11 increases the caspase-3 activity specifically in PHA-stimulated Jurkat cells. Caspase-3 activity assays were performed using fluorogenic Ac-DEVD-AFC. Cells were then incubated for 24 h with 0, 1, or 10 µg/mL PHA and 0 to 1 μM P11 in complete culture medium. Caspase-3 activity was about twofold increased at 10 µg/mL PHA stimulation for 24 h. Moreover, the addition of 10 nM compound P11 to PHA-stimulated Jurkat cells already produced a maximal activation of caspase-3. Statistically significant differences are indicated by: ns = non-significant, * *p* < 0.05, ** *p*< 0.01, *n* = 6.

As shown in Figure 6, compound P11 alone, even at a concentration of 1 µM, did not induce any increase in capase-3 activity. However, caspase-3 activity was increased by ≈ twofold after 10 µg/mL PHA stimulation during 24 h (1836 ± 132 AU vs. 1037 ± 100 AU, *n* = 5). Interestingly, the additional presence of 10 nM compound P11 already induced a maximal activating effect on caspase-3 activity with a ≈ threefold increase (3332 ± 113 AU at 1 µM, *n* = 5). 

**Figure 7:**
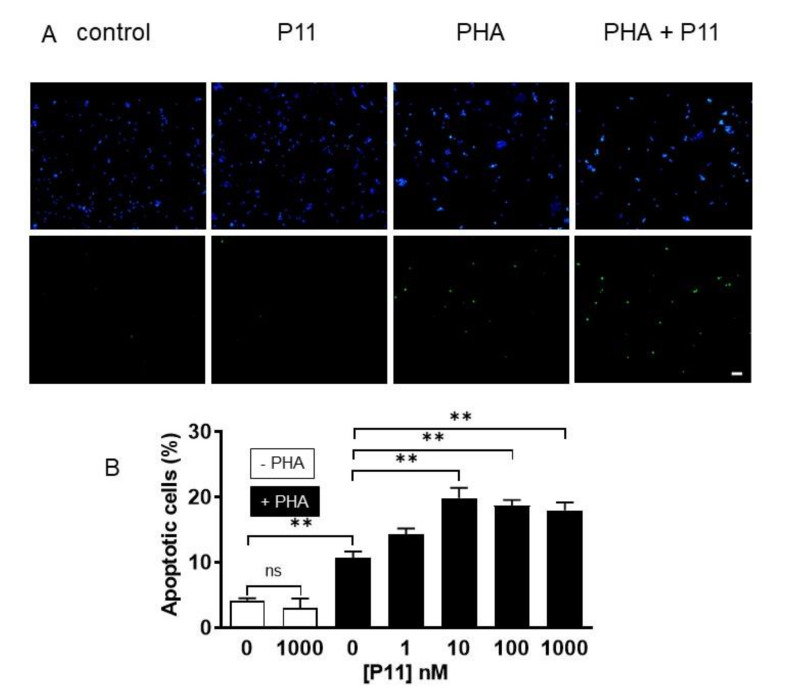
Compound P11 increases DNA fragmentation in PHA-stimulated Jurkat cells. (**A**) Cells were stained with DAPI (blue, top) to visualize the nuclei and with the TUNEL method (green, bottom) to visualize apoptotic cells. Each staining was merged with the phase contrast image to visualize whole cells. In control conditions or after stimulation by 1 µM P11 for 24 h, only few apoptotic cells were detectable (~5%). PHA (10 µg/mL)-stimulated cells demonstrated a significant increase in apoptotic cells. Costimulation with concentrations > 10 nM of P11 compound induced a ≈ 1.5-fold increase. Representative pictures of cells after 24 h of treatment with 10 µg/mL PHA alone or with 1–1000 nM P11, or with 1 µM P11 alone. Scale bar = 50 µm. (**B**) The number of apoptotic cells (ratio of TUNEL-positive cells/DAPI-positive cells, expressed in percent) was counted after 24 h of treatment. Statistically significant differences are indicated by: ns = non-significant, ** *p* < 0.01, *n* = 6.

Moreover, similar results were obtained when the percentage of dead cells was measured with the TUNEL assay (Figure 7). In resting conditions, less than 5% of the cells had fragmented DNA (4.2 ± 0.3, *n* = 3). PHA alone induced a threefold increase in the number of cells with fragmented DNA (13.3 ± 1.6%, *n* = 3). At concentrations > 10 nM, compound P11 induced a ≈ 4.5-fold increase (17.9 ± 1.2%, *n* = 3 at 1 µM).

Taken together, these results demonstrate that compound P11 is not toxic for the cells when added alone but could profoundly impair Jurkat cell activation by inducing their apoptosis. 

## 3. Discussion

Since the discovery of the molecular partners implied in SOCE in the middle of the 2000s, the characterization of their modulators has emerged as attracting enough for pharmaceutical companies like GSK and Hoffmann-La Roche or newcomers like Calcimedica. Indeed, it appeared that aberrant SOCE could be associated with autoimmune disorders, inflammation, or cancers [23]. Inhibiting SOCE appears as a new and promising way to treat this kind of disease or to ease it.

SOCE was functionally characterized during the 1980s (formerly named capacitative calcium entry by Jim Putney [24]), but its molecular mechanism was only unveiled in the last decade [3,25]. After store depletion, the ER proteins STIM1 interact with and activate the plasma membrane Ca^2+^ channels mainly consisting of Orai1 proteins [3]. Noteworthy, loss-of-function mutations in the Orai1 gene, rendering the Orai1 protein unfunctional or absent in the plasma membrane, via SOCE inhibition, mainly impair the immune system [26]. On the opposite, some gain-of-function mutations in Orai1 or STIM1, allowing a constitutive SOCE or an increased SOCE amplitude are also associated with diseases like the York platelet syndrome or the Stormorken syndrome [27]. From this fact, the control of Orai1 channels (directly or indirectly via STIM1) and the SOCE emerged as a new way to control the immune system, but also the proliferation of cancerous cells or other diseases. Noteworthy, two Orai1 paralogues exist, Orai2 and Orai3 with “less global” roles: thus, Orai2 regulates the SOCE amplitude by forming heteromeric channels with Orai1 with reduced conductance in some T cells [28]. About Orai3; there exists a dichotomy between SOCE observed in breast cancer cell lines and in cells from patient samples. In estrogen-receptor expressing cells, Orai3 channels are responsible for the SOCE, while Orai1 are the ones responsible in estrogen-receptor-negative cells [29]. Thus Orai3 channels could also represent a target of interest for the treatment of the estrogen-receptor-positive breast cancer cells which represent 80% of breast cancers [29]. 

However, in the vast majority of the cell types, the spatial emplacement of the three Orai proteins in the cells seem to be different: Orai1 mainly is located in the plasma membrane [3,30], Orai2 in the internal compartments, and Orai3 in the ER membrane [31,32,33]. Thus, in T and B cells only Orai1 participates in SOCE [3,9,19]. 

Despite huge therapeutic potential, the characterization of new SOCE modulators is impaired by the limited knowledge of the Orai1 structure. Indeed, only the crystal structure of the Drosophila melanogaster dOrai, which shares 73% sequence homology with hOrai1, has been published at a 3.35 Å resolution [34]. Furthermore, no hOrai1 structure has yet been established when bound to STIM1 in its active conformation. A controversy also still exists on the number of Orai proteins needed to form a functional channel, tetramer [35] or hexamer [36,37], although the latter seems to be the most accepted currently. Therefore, the development of new SOCE modulators is still not possible by docking work but powered by the characterization of analogs of existing modulators.

Among SOCE modulators, we focused our work on 2-APB, based on its following three properties: (i) 2-APB is a small molecule; (ii) 2-APB potentiates SOCE amplitude at low concentrations (≈ 5 µM) and inhibits SOCE at higher concentrations (> 30 µM); (iii) the relative ease to synthesize analogs. In three previous studies, we already showed that the central boron-oxygen-carbon plays a role in the potentiation capacity and the ethanolamine and the two phenyl groups in the inhibition [7,9,38]. Thus, by modifying one or the other part of the 2-APB molecule, it is possible to create analogs with better potentiation or inhibition ability. Furthermore, our 2-APB analogs request Orai1 expression to act on the T cell SOCE [9].

In the present work, we only modified the two phenyl rings by adding simple radicals like methyl or by replacing them with more complex cycles to develop new SOCE inhibitors (Figure 1). Interestingly, all our molecules modified on the two phenyl groups lost their potentiation capacity. In Djillani et al. 2014 [7], we showed that two analogs where one (nicknamed “methoxy-APB”) or two methoxyl (“dimethoxy-APB”) groups were added on the same phenyl, keeping the second one intact, still had a potentiation capacity (even if it was decreased). On the opposite, our new compounds with added methoxyl groups on the phenyl groups have only inhibiting properties, confirming that increasing the size of both phenyl groups allows the creation of new SOCE inhibitors. 

Among our new molecules, compound P11 was the most potent SOCE inhibitor. With an inhibitory constant between 32 and 75 nM depending on the cell type used and a complete SOCE inhibition at a concentration well below 1 µM, P11 is one of the best SOCE inhibitors described in the literature. The twofold difference in *K*i between cell types could reflect a difference in the Store-Operated Calcium Channels (SOCC) composition. Indeed, it has been shown that the expression ratio of Orai1/STIM1 modulates the sensitivity of the SOCE to 2-APB [39]. Furthermore, Orai1 can be associated with some TRP channel subunits like TRPC1, TRPC3, and TRPC6 [40] which can also interact with 2-APB, and thus potentially with its analogs. 

The free rotation between the two phenyl groups on the same side seems to play an important role in the inhibition capacity of compound P11. When this rotation is impaired by a second carbon “bridge,” like in compound P24, the molecule was not able to inhibit, even partially, the SOCE at concentrations below 1 µM. Thus, we assume that when 2-APB or its analogs enter the SOCC pore, the two “wings” (phenyl or larger/longer aryls) need to tether and reach the hypothetic inhibitory binding site(s).

However, the great advantage of compound P11 is that it avoids the major side effects of 2-APB, as it did neither inhibit the IP_3_R nor the SERCA pumps of the ER. Furthermore, it did not induce a Ca^2+^ leak from the ER by an unknown pathway, for example, as our compound P8 or as DPB162-AE [10] did. This absence of side effects could be related to the apparent non-toxicity of the molecule on resting cells: compound P11 by itself at 1 µM did neither increase DNA fragmentation nor cell death.

Jim Putney showed more than 30 years ago that the Ca^2+^ influx was activated by the ER depletion, and that this Ca^2+^ entry he called “capacitative calcium entry,” eventually named store-operated Ca^2+^ entry (SOCE), allowed the refilling of the ER [24]. More recently, Zheng et al. [41] showed that the refilling of ER Ca^2+^ is fully impaired in cells that do not express all three Orai isoforms. However, after 24 h in culture, Ca^2+^ stores became eventually refilled, meaning that SOCE is critical for the immediate refilling of the ER Ca^2+^ stores. It is known that ER Ca^2+^ store depletion leads to ER stress induction that, if not counteracted by the unfolded protein response, will provoke apoptosis. In our experiments, the cells were stimulated with PHA and different [P11] for 24 h to study the apoptosis induction and to measure IL-2 synthesis and caspase-3 activity. Remarkably, P11 was not by itself cytotoxic, and it exerted a cytotoxic effect only when the cells were stimulated and the [Ca^2+^]_cyt_ rise impaired (Figure 5A). Surprisingly P11 induced a decrease of [Ca^2+^]_cyt_ beneath pre-stimulation values. As the cells were still under PHA stimulation, we assume that there was a constant ER Ca^2+^ release and that the SOCE blockade impaired the refilling of the ER Ca^2+^ content and could induce an ER stress leading to the apoptosis.

Enlarging the 2-APB molecule on the two phenyl groups allows the development of increasingly efficient inhibitors. We anticipate that our compound P11 could also be further modified and are confident that molecules with an inhibition capacity in the low nanomolar range could be obtained. 

Thus, from our studies and those of Mikoshiba’s group, it appears possible to create 2-APB analogs with higher potency and selectivity. Due to its dual effect on SOCE, the advantage of 2-APB over other SOCE modulators is that, depending on the substitution performed, it is possible to create inhibiting or potentiating agents. Our compound P11 could, therefore, represent a new promising branch of the 2-APB family that could help to cure human disorders in which Ca^2+^ homeostasis is modified.

## 4. Materials and Methods

### 4.1. Cell Lines

Jurkat (acute T cell leukemia from ATCC), DG75 (Burkitt B lymphoma, kind gift of Martin Rowe, University of Birmingham, UK), and U937 (monocytic cell line from ATCC) cell lines were basically maintained in RPMI-1640 medium (Lonza, Levallois-Perret, France) supplemented with 10% heat-inactivated fetal calf serum and 2 mM L-glutamine, at 37 °C in a 5% CO_2_ humidified atmosphere. The MDA-MB231 breast cancer cell line (form ATCC) was maintained in same conditions as the leukocyte cell lines except that DMEM (Lonza, Levallois-Perret, France) medium was used instead of RPMI1640. Epstein-Barr virus immortalized B cells from Orai1-deficient and healthy patients were a kind gift of Capucine Picard and Alain Fischer (study center of primary immunodeficiencies, AP-HP, Hopital Necker, Paris France [19]) and maintained as the leukocytes cell lines. The L15 fibroblast cell line stably overexpressing IP_3_R1 [42] was a generous gift of Katsuhiko Mikoshiba (now at Shanghai Tech Univ., China). The cells were maintained in DMEM medium (Gibco^®^, Life Technologies, Gent, Belgium) supplemented with 10% heat-inactivated fetal calf serum, 3.8 mM L-glutamine, 1% non-essential amino acids, 85 IU mL^−1^ penicillin, 85 µg/mL streptomycin, 400 µg/mL geneticin, and 25 mM Na-Hepes (pH 7.4) at 37 °C and 10% CO_2_.

### 4.2. Cytosolic Ca^2+^ Concentration and SOCE Measurement

[Ca^2+^]_cyt_ was recorded by a fluorimetric ratio technique as previously described [7,43,44]. Leukocytes were spun and resuspended at a density of 10^6^ cells/mL in Hepes buffered saline (HBS; 135 mM NaCl, 5.9 mM KCl, 1.2 mM MgCl_2_, 11.6 mM Hepes, 11.5 mM glucose adjusted to pH 7.3 with NaOH). Due to the presence of 0.002% Ca^2+^ in the NaCl, this HBS medium contained 0.1 mM of Ca^2+^. Then, cells were incubated in the dark with 4 μM Indo-1-AM for one hour at room temperature under slow agitation. Cells were then centrifuged and resuspended in HBS medium before measurement. To perform the experiments, 0.5 to 1 × 10^6^ cells were suspended in 2 mL HBS in a quartz cuvette and inserted into a spectrofluorophotometer (RF-1501 Shimadzu Corporation, Kyoto, Japan) connected to a PC computer (Dell Computer Corp., Montpellier, France). A temperature of 37°C was maintained by circulating water from a bath. Ultraviolet light of 360 nm was used for excitation of Indo-1, and emissions at 405 and 480 nm were recorded. Background and autofluorescence of the cell suspension were subtracted from the recordings. The maximum Indo-1 fluorescence (*R*_max_) was obtained by adding 1 µM ionomycin to the cell suspension in the presence of 10 mM extracellular CaCl_2_. Minimum fluorescence was determined following the depletion of external Ca^2+^ with 5 mM EGTA. [Ca^2+^]_cyt_ was calculated according to the equation [Ca^2+^]_cyt_ = *K*_d_ (R-*R*_min_)/(*R*_max_-R), where *K*_d_ is the apparent dissociation constant of Indo-1-calcium complex (230 nM), and R is the ratio of fluorescence values recorded at 380 nm in absence and presence of 10 mM CaCl_2_ [43]. 

To visualize the SOCE, cells were placed into the quartz cuvette and 0.5 EGTA was added just before the beginning of the recordings. To induce the SOCE, the cells were treated with 1 μM thapsigargin (TG) during 10 min in Ca^2+^-free HBS to induce Ca^2+^ release from the ER and the opening of SOCE channels [7,45]. Then, 1 mM CaCl_2_ was added to measure the change in [Ca^2+^]_cyt_ subsequently to Ca^2+^ influx [45]. In the dose-response experiments, different concentrations of 2-APB and its analogs were added 30 s prior to Ca^2+^ readdition [7,9]. 

In experiments with PHA, cells were placed in the cuvette, and 0.9 mM CaCl_2_ was added to reach a 1 mM extracellular concentration. Free Ca^2+^ concentrations were calculated using the Bad4 software [46]

### 4.3. Measurement of Mn^2+^ Influx by Indo-1 Quenching

To study directly the divalent ion influx through the SOCC with selected 2-APB analogs, we performed Mn^2+^ quenching experiments. Mn^2+^ ions bind to Indo-1 and quench the 430 nm emission wavelength fluorescence after excitation at 360 nm. The decrease of fluorescence is directly dependent on Mn^2+^ entry through SOCC. For measurements, cells were treated for 10 min with 1 µM TG to open SOCC, whereupon 100 µM MnCl_2_ was added instead of CaCl_2_. After 60 s a 2-APB analog was added as indicated and the change in the curve slope was measured.

### 4.4. Unidirectional 45Ca^2+^ Flux Experiments

The L15 fibroblasts stably overexpressing IP_3_R1 were seeded in gelatin-coated 12-well plates (Greiner, Frickenhausen, Germany) at a density of 60,000 cells per well. Unidirectional ^45^Ca^2+^ flux experiments on confluent cell monolayers were performed at 30 °C after membrane permeabilization with saponin (40 µg/mL), exactly as previously described [9,47]. After permeabilization, the non-mitochondrial Ca^2+^ stores were loaded for 45 min in 120 mM KCl, 30 mM imidazole–HCl (pH 6.8), 5 mM MgCl_2_, 5 mM ATP, 0.44 mM EGTA, 10 mM NaN_3,_ and 150 nM free ^45^Ca^2+^ (28 µCi mL^−1^). Subsequently, efflux was initiated by incubation in efflux medium (120 mM KCl, 1 mM EGTA, 10 µM TG and 30 mM imidazole–HCl pH 6.8). The latter medium was replaced every two minutes for 18 minutes. The exact amount of Ca^2+^ at each time point is calculated by summing in retrograde order the amount of radioactivity remaining in the cells at the end of the efflux and the radioactivity collected during the successive time intervals. This allows to calculate the steady-state content of the ER Ca^2+^ stores (indicative for the SERCA activity, after subtraction of the loading achieved in the presence of 2 µM TG) the basal leak rate of Ca^2+^ out of the stores and the IP_3_-induced Ca^2+^ release after addition of IP_3_ (0.7 µM) [14]. These various parameters were measured both in control conditions or in the presence of various 2-APB analogs as indicated. To assess the effect of the 2-APB analogs on the SERCA-mediated loading of the ER Ca^2+^ stores, the analogs were added during the ^45^Ca^2+^-loading phase as described previously [9]. Obviously, compounds that strongly stimulated the basal Ca^2+^ leak out of the stores (as P8) could not be investigated for their effect on SERCA activity (as Ca^2+^ loading was already compromised by the increased leak) or on IP_3_-induced Ca^2+^ release (as the decreased luminal Ca^2+^ level impacted both the driving force for Ca^2+^ release and the sensitivity of the IP_3_R) [48].

### 4.5. Interleukin-2 (IL-2) Assay

Forty-eight-well plates were seeded with 10^6^ Jurkat T cells per well. Cells were then incubated for 24 h with or without 10 µg/mL PHA and 0 to 1 µM P11 in complete culture medium. After 24 h, supernatants were collected, and IL-2 amounts were quantified using a Quantikine Human IL-2 Immunoassay (R&D Systems Europe, Lille, France). Optical density (OD) was measured at 450 nm using a Victor3 plate reader (Perkin Elmer, Courtaboeuf, France). The concentration of supernatant IL-2 was determined from the OD curve obtained with the standard. Results were the mean of four repeats. In parallel plates, cell viability was accessed after treatment with trypan blue and counting with a Malassez hematimeter (Sigma-Aldrich, Saint Quentin Fallavier, France).

### 4.6. Fluorometric Assay for Caspase-3 Activity

Caspase-3 activity was measured using the fluorogenic substrate Ac-DEVD-AFC (Tebu-bio, Le Perray-en-Yvelines, France) according to the manufacturer’s instructions. Briefly, 48-well plates were seeded with 10^6^ Jurkat T cells per well. Cells were then incubated for 24 h with 0, 1, or 10 µg/mL PHA and 0 to 1 µM P11 in complete culture medium. After 24 h, cells were collected and lysed in RIPA lysis buffer supplemented with protease inhibitor cocktails (Life Science Biorad, Marnes la Coquette, France). The protein samples were quantified using the DC protein assay reagent (Biorad, Les Ulis, France). Cell lysates (50 µg) were diluted with reaction buffer (200 mM Hepes pH 7.4, 1 mM EDTA, 20% sucrose and 20 mM dithiothreitol) and incubated with the fluorogenic substrate (20 µM final concentration) for 45 min up to 7 h at 37 °C. The release of 7-amino-4-trifluoromethylcoumarin (AFC) was measured by fluorescence (excitation at 405 nm, emission at 505 nm) using a Victor3 plate reader (Perkin Elmer, Courtaboeuf, France).

### 4.7. Apoptosis Detection by the Terminal Transferase dUTP Nick End Labelling (TUNEL) Method 

We measured the apoptosis levels in Jurkat cells by the “in situ cell death detection” kit (Roche Applied Science, Meylan, France). Briefly, 50,000 cells/well were seeded in a 96-well plate and treated with or without 10 µg/mL PHA and 0 to 1 µM P11 for 6, 12, or 24 h. Cells were spun, fixed, and permeabilized according to the manufacturer’s instructions and the terminal transferase dUTP Nick end labelling reaction was performed in the dark during 60 min at 37 °C. For visualization of the results, an epifluorescence microscope (Axioskop, Karl Zeiss, Le Pecq, France) was used at an excitation wavelength of 488 nm, and an emission wavelength of 545 nm. Cells were also loaded with 4’,6’ –diamidino-2-phenylindole (DAPI) to visualize the nuclei. Total cell number (>200) and TUNEL-positive cells were counted in several fields (>5) and the ratio of apoptotic cells was calculated. This experiment was repeated three times.

### 4.8. Chemicals

TG and ionomycin were purchased from Calbiochem-Merck (Nottingham, UK). 

Synthesis of 2-APB and its analogs were done using previously described methodologies [49]. General procedure for APB analog synthesis: To a solution of arylbromide (3 eq), magnesium (3.5 eq), and di*iso*propylamine borane complex (DIPAB, 1 eq) in dry THF (1 mL/mmol) was added at room temperature to a solution of PhMgBr (2 M in THF, 0.05 eq). After 10 min, (or the end of gas evolution on large scale), the reaction mixture was heated to 70°C until no arylbromide remained in the reaction mixture (8 to 16 h) as witnessed using TLC monitoring. At 0 °C, 1 mL/mmol of dry MeOH was added (caution, exothermic reaction). After 1 h, the reaction mixture was concentrated under reduced pressure, and diluted in a mixture of 1N HCl/MeOH (7:3, 10 mL/mmol), after 1 h, the product was extracted with Et_2_O (3 × 10 mL/mmol) and the combined organic phases washed with 1N HCl (10 mL/mmol) and brine (3 × 10 mL/mmol). Organic phases were then dried over anhydrous Na_2_SO_4_ and concentrated under reduced pressure. The residue was dissolved in Et_2_O (4 mL/mmol) and 1.2 eq of ethanolamine was added. Depending on substituents, precipitation occurs spontaneously or requires addition of pentane. Crystals were filtered, washed with pentane, and dried under vacuum to yield the pure borinic acid 2-aminoethyl ester. 

DIPAB Diisopropylamine-borane complex (55124-35-1)

A 3000 mL three-necked round-bottomed flask equipped with a mechanical stirrer, a thermometer, and a dropping funnel was charged with THF (1500 mL) and NaBH_4_ (56.75 g, 1.5 mol) The heterogeneous mixture was vigorously agitated using a mechanical stirrer and cooled with an ice/salt bath below 0 °C. The dropping funnel was charged with 37.8 mL of H_2_SO_4_ (0.71 mol) The H_2_SO_4_ was added dropwise maintaining the internal temperature below −5 °C over 3 h. An iPr_2_NH (140 mL, 1 mol) solution in THF (100 mL) was added dropwise maintaining the temperature below 0 °C over 1 h 30 m. The mixture was vigorously agitated during 20 h at room temperature. The mixture was filtrated over a N°3 fritted funnel and the resulting solid was triturated with THF (3 × 400 mL). THF filtrate was concentrated under reduced pressure the residue was taken with CH_2_Cl_2_, and then filtrated to eliminate all solid residues. The filtrate was washed with water (4 × 200 mL). The organic phase was dried over Na_2_SO_4_ and concentrated under reduced pressure to give DIPAB as colorless oil which solidified upon cooling (102 g, 90%). ^1^H NMR (400 MHz, CDCl_3_): δ (ppm) 2.96 (s, 1H), 2.89–2.75 (m, 2H), 1.95–1.48 (m, 13H), 1.3–1.01 (m, 7H). ^11^B NMR (128 MHz, CDCl_3_): δ (ppm)−20.32 (q).

P1, 2-APB, ((diphenylboryl)oxy)ethanamine (15614-89-8) [50] 

1.02 g of ((diphenylboryl)oxy)ethanamine were obtained following the general procedure using 1.73 g of bromobenzene as a white solid (92% yield). ^1^H NMR (300 MHz, DMSO-*d*_6_): *δ* (ppm) 7.40 (d, *J* = 7.2 Hz, 4H), 7.13 (t, *J* = 7.2 Hz, 4H), 7.03 (t, *J* = 7.2 Hz, 2H), 6.07 (bt, 2H), 3.77 (t, *J* = 6.35 Hz, 2H), 2.90 – 2.75 (p, *J* = 6.35 Hz, 2H). ^11^B NMR (96 MHz, DMSO-*d*_6_): *δ* (ppm) 4.1 (s). ^13^C NMR (75 MHz, DMSO-*d*_6_): *δ* (ppm) 131.5, 126.6, 124.9, 62.4, 41.3. Melting point: 192.5 °C. 

P2, 2-((bis(4-methoxyphenyl)boryl)oxy)ethanamine (37763-62-5) [50]

1.21 g of 2-((bis(4-methoxyphenyl)boryl)oxy)ethanamine were obtained following the general procedure H using 2.06 g of 3-bromoanisole as a white solid (81% yield). ^1^H NMR (300 MHz, CDCl_3_): *δ* (ppm) 7.26 (d, *J* = 8.5 Hz, 4H), 6.72 (d, *J* = 8.5 Hz, 4H), 5.88 (bt, 2H), 3.74 (t, *J* = 6.35 Hz, 2H), 3.67 (s, 6H), 2.80 (t, *J* = 6.35 Hz, 2H). ^11^B NMR (96 MHz, DMSO-*d*_6_): *δ* (ppm) 10.3 (s). ^13^C NMR (101 MHz, DMSO-*d*_6_): *δ* (ppm) 148.2, 133.3, 131.6, 127.3, 62.4, 41.3, 20.9. Melting point: 181.7 °C. LC-MS (ESI): C_16_H_20_BNO_3_ 570.70 [2M+H]^+^.

P3, 2-((di-p-tolylboryl)oxy)ethanamine (19565-45-8) [51] 

1.04 g of 2-((di-p-tolylboryl)oxy)ethanamine were obtained following the general procedure using 1.88 g of 4-bromotoluene as a white solid (90% yield). ^1^H NMR (300 MHz, DMSO-*d*_6_): *δ* (ppm) 7.25 (d, *J* = 7.7 Hz, 4H), 6.94 (d, *J* = 7.7 Hz, 4H), 5.92 (bt, 2H), 3.73 (t, *J* = 6.4 Hz, 2H), 2.80 (t, *J* = 6.4 Hz, 1H), 2.20 (s, 6H). ^11^B NMR (96 MHz, DMSO-*d*_6_): *δ* (ppm) 5.7 (s) ^13^C NMR (75 MHz, DMSO-*d*_6_): *δ* (ppm) 148.2, 133.3, 131.6, 127.3, 62.4, 41.3, 20.9. Melting point: 185.3 °C. LC-MS (ESI): C_16_H_20_BNO [2M+H]^+^: 506.80.

P4, 2-((bis(3-methoxyphenyl)boryl)oxy)ethanamine

1.12 g of 2-((bis(3-methoxyphenyl)boryl)oxy)ethanamine were obtained following the general procedure using 2.06 g of 3-bromoanisole as a white solid (83% yield). ^1^H NMR (300 MHz, DMSO-*d*_6_): *δ* (ppm) 7.06 (t, *J* = 7.8 Hz, 2H), 6.96 (m, 4H), 6.60 (dd, *J* = 7.8, 1.7 Hz, 2H), 6.05 (bt, 2H), 3.76 (t, *J* = 6.4 Hz, 2H), 3.68 (s, 6H), 2.81 (p, *J* = 6.4 Hz, 2H). ^11^B NMR (96 MHz, DMSO-*d*_6_): *δ* (ppm) 5.3 (s). ^13^C NMR (75 MHz, DMSO-*d*_6_): *δ* (ppm) 158.2, 127.6, 123.9, 116.9, 110.1, 62.4, 54.5, 41.3. Melting point: 137.7 °C. LC-MS (ESI): C_16_H_20_BNO_3_ 570.70 [2M+H]^+^.

P5, 2-((di-m-tolylboryl)oxy)ethanamine (97979-11-8) [52] 

1.05 g of 2-((di-m-tolylboryl)oxy)ethanamine were obtained following the general procedure using 1.88 g of 3-bromotoluene as a white solid (82% yield). ^1^H NMR (300 MHz, DMSO-*d6*): *δ* (ppm) 7.26–7.10 (m, 4H), 7.01 (t, J = 7.4 Hz, 2H), 6.84 (d, J = 7.4 Hz, 2H), 6.00 (bt, 2H), 3.74 (t, J = 6.4 Hz, 2H), 2.80 (p, J = 6.4 Hz, 2H), 2.22 (s, 6H) ^11^B NMR (96 MHz, DMSO-*d6*): δ (ppm) 4.8 (s). ^13^C NMR (75 MHz, DMSO-d6): δ (ppm) 135.2, 132.7, 129.1, 126.9, 125.9, 62.80, 41.8, 21.9. Melting point: 193.4 °C LC-MS (ESI): C_16_H_20_BNO [2M+H]+: 506.8.

P6, 2-((di(naphthalen-2-yl)boryl)oxy)ethanamine (515157-62-7) [53]

1.50 g of 2-((di(naphthalen-2-yl)boryl)oxy)ethanamine were obtained following the general procedure using 2.28 g of 2-bromonaphtalene as a white solid (92% yield). ^1^H NMR (300 MHz, DMSO-*d*_6_): *δ* (ppm) 7.95 (s, 2H), 7.76 (d, *J* = 7.7 Hz, 4H), 7.73–7.61 (m, 4H), 7.44–7.30 (m, 4H), 6.34 (bt, 2H), 3.88 (t, *J* = 6.3 Hz, 2H), 3.02–2.88 (p, *J* = 6.3 Hz, 2H). ^11^B NMR (96 MHz, DMSO-*d*_6_): *δ* (ppm) 6.0 (s). ^13^C NMR (75 MHz, DMSO-*d*_6_): *δ* (ppm) 132.9, 131.9, 130.8, 129.7, 127.4, 127.2, 125.4, 124.9, 124.3, 62.6, 41.6. Melting point: 206.2 °C. LC-MS (ESI): C_22_H_20_BNO 650.80 [2M+H]. 

P7, 2-((bis(3,4-dimethylphenyl)boryl)oxy)ethanamine (99269-70-2)

1.11 g of 2-((bis(3,4-dimethylphenyl)boryl)oxy)ethanamine were obtained following the general procedure using 2.04 g of 4-bromo-*o*-xylene as a white solid (79% yield). ^1^H NMR (300 MHz, DMSO-*d*_6_): *δ* (ppm) 7.12 (s, 2H), 7.06 (d, *J* = 7.4 Hz, 2H), 6.87 (d, *J* = 7.4 Hz, 2H), 5.87 (bt, 2H), 3.71 (t, *J* = 6.3 Hz, 2H), 2.78 (p, *J* = 6.3 Hz, 2H), 2.13 (s, 6H), 2.12 (s, 6H). ^11^B NMR (96 MHz, DMSO-*d*_6_): *δ* (ppm) 5.9. ^13^C NMR (100 MHz, DMSO-*d*_6_): *δ* (ppm) 133.4, 133.1, 131.9, 129.2, 127.8, 62.30, 41.3, 19.7, 19.2. Melting point: 209.2 °C. LC-MS (ESI): C_18_H_24_BNO 562.75 [2M+H]^+^.

P8, 2-((bis(4-(tert-butyl)phenyl)boryl)oxy)ethanamine (1379680-33-7)

1.24 g of 2-((bis(4-(tert-butyl)phenyl)boryl)oxy)ethanamine were obtained following the general procedure using 2.34 g of 4-terbutyl-bromobenzene as a white solid (85% yield). ^1^H NMR (300 MHz, DMSO-*d*_6_): *δ* (ppm) 7.32 (d, *J* = 8.2 Hz, 4H), 7.15 (d, *J* = 8.2 Hz, 4H), 5.98 (bt, 2H), 3.75 (t, *J* = 6.25 Hz, 2H), 2.80 (p, *J* = 6.25 Hz, 2H), 1.23 (s, 18H). ^11^B NMR (96 MHz, DMSO-*d*_6_): *δ* (ppm) 6.1 (s). ^13^C NMR (100 MHz, DMSO-*d*_6_): *δ* (ppm) 146.7, 131.2, 123.3, 62.4, 41.4, 33.9, 31.4. Melting point: 255.5 °C. LC-MS (ESI): C_22_H_32_BNO 674.90 [2M+H]^+^.

P9, 2-(bis(4-(tert-butyl)phenyl)boryl)oxy)ethanamine

1.48 g of 2-(bis(4-(tert-butyl)phenyl)boryl)oxy)ethanamine were obtained following the general procedure using 2.34 g of 4-*n*-butyl-bromobenzene as a white solid. (70% yield). ^1^H NMR (400 MHz, DMSO-*d*_6_): *δ*(ppm) 7.29 (d, *J* = 7.7 Hz, 4H), 6.95 (d, *J* = 7.7 Hz, 4H), 5.96 (bt, 2H), 3.75 (t, *J* = 6.4 Hz, 2H), 2.88–2.77 (p, *J* = 6.4 Hz, 2H), 2.58–2.42 (m, 4H), 1.51 (m, 4H), 1.30 (m, 4H), 0.89 (t, *J* = 7.4 Hz, 6H). ^11^B NMR (128 MHz, DMSO-*d*_6_): *δ* (ppm) 5.8 (s). ^13^C NMR (100 MHz, DMSO-*d*_6_): *δ* (ppm) 148.6, 138.4, 131.5, 126.6, 62.4, 41.3, 34.8, 33.5, 21.8, 13.8. Melting point: 99.0 °C. LC-MS (ESI): C_22_H_32_BNO 674.85 [2M+H]^+^.

P10, 2-((bis(3,5-dimethylphenyl)boryl)oxy)ethanamine

1.02 g of 2-((bis(3,5-dimethylphenyl)boryl)oxy)ethanamine were obtained following the general procedure using 2.04 g of 4-bromo-*m*-xylene as a white solid (85% yield). ^1^H NMR (300 MHz, DMSO-*d*_6_): *δ* (ppm) 6.97 (s, 4H), 6.65 (s, 2H), 5.92 (bt, 2H), 3.70 (t, *J* = 6.25 Hz, 2H), 2.78 (p, *J* = 6.25 Hz, 2H), 2.18 (s, 12H). ^11^B NMR (96 MHz, DMSO-*d*_6_): *δ* (ppm) 5.6 (s). ^13^C NMR (100 MHz, DMSO-*d*_6_): *δ* (ppm) 134.6, 129.4, 126.3, 62.3, 41.3, 21.3. Melting point: 294.5 °C. LC-MS (ESI): C_18_H_24_BNO 562.75 [2M+H]^+^.

P11, 2-(dibiphenyl-4-ylboryloxy)ethanamine (102032-41-7)

1.55 g of 2-(dibiphenyl-4-ylboryloxy)ethanamine were obtained following the general procedure using 2.56 g of 4-bromobiphenyl as a white solid (79 % yield). ^1^H NMR (300 MHz, DMSO-*d*_6_): *δ* (ppm) 7.60 (d, *J* = 7.3 Hz, 4H), 7.55 (d, *J* = 8.0 Hz, 4H), 7.46 (d, *J* = 7.3 Hz, 4H), 7.41 (d, *J* = 8.0 Hz, 2H), 7.30 (t, *J* = 7.3 Hz, 2H), 6.19 (bt, 2H), 3.83 (t, *J* = 6.4 Hz, 2H), 2.95–2.83 (p, *J* = 6.4 Hz, 2H). ^11^B NMR (96 MHz, DMSO-*d*_6_): *δ* (ppm) 7.8 (s). ^13^C NMR (100 MHz, DMSO-*d*_6_): *δ* (ppm) 151.0, 141.3, 137.0, 132.2, 128.8, 126.7, 126.4, 125.1, 62.6, 41.5. Melting point: 232.0 °C. LC-MS (ESI): C_26_H_24_BNO 754.80 for [2M+H]^+^.

P12, 2-(dibenzo[b]thiophen-5-ylboryloxy)ethanamine

1.34 g of 2-(dibenzo[b]thiophen-5-ylboryloxy)ethanamine were obtained following the general procedure using 2.34 g of 5-bromobenzo[b]thiophene as a white solid (68 % yield). ^1^H NMR (300 MHz, DMSO-*d*_6_): *δ* (ppm) 7.92 (s, 2H), 7.75 (d, *J* = 8.1 Hz, 2H), 7.55 (d, *J* = 5.4 Hz, 2H), 7.47 (d, *J* = 8.1 Hz, 2H), 7.33 (d, *J* = 5.4 Hz, 2H), 6.21 (bt, 2H), 3.85 (t, *J* = 6.4 Hz, 2H), 2.98–2.85 (p, *J* = 6.4 Hz, 2H). ^11^B NMR (96 MHz, DMSO-*d*_6_): *δ* (ppm) 6.7 (s) ^13^C NMR (100 MHz, DMSO-*d*_6_): *δ* (ppm) 147.3, 138.9, 136.4, 128.8, 126.4, 125.2, 124.0, 120.5, 62.6, 41.5. Melting point: 228.0 °C. LC-MS (ESI): C_18_H_16_BNOS_2_ 674.55 [2M+H]^+^.

P13, 2-(bis(4-chlorophenyl)boryl)oxy)ethanamine (61733-90-2)

1.132g of 2-(bis(4-chlorophenyl)boryl)oxy)ethanamine were obtained as a white solid starting from 575 mg of diisopropylamine borane complex following the general procedure (80%). ^1^H NMR (300 MHz, DMSO-*d_6_*): *δ* (ppm) 7.38–7.35 (dd, *J* = 9Hz, 4H), 7.19–7.16 (t, *J* = 9Hz, 4H), 6.18 (brs, 2H), 3.77–3.73 (t, *J* = 6Hz, 2H), 2.87–2.79 (p, *J* = 6Hz, 2H). ^11^B NMR (100 MHz, DMSO-*d_6_*): *δ* (ppm) 4.0. ^13^C NMR (75 MHz, DMSO-*d_6_*): *δ* (ppm) 133.8, 130.5, 129.1, 127.0, 62.9, 41.8.

P14, 2-(bis(4-fluorophenyl)boryl)oxy)ethanamine (176913-70-5)

871 mg of 2-(bis(4-fluorophenyl)boryl)oxy)ethanamine were obtained as a pale yellow solid starting from 575 mg of diisopropylamine borane complex following the general procedure (80%). ^1^H NMR (300 MHz, DMSO-*d_6_*): *δ* (ppm) 7.40-7.36 (t, *J* = 6Hz, 4H), 6.98–6.92 t, *J* = 9Hz, 4H), 6.11 (brs, 2H), 3.77 (t, *J* = 6Hz, 2H), 2.84 (p, *J* = 6 Hz, 2H). ^11^B NMR (100 MHz, CDCl_3_): *δ* (ppm) 3.9. ^13^C NMR (75 MHz, CDCl_3_): *δ* (ppm) 163.0, 159.8, 133.6, 113.5, 62.9, 41.8.

P16, 2-(((4-methoxyphenyl)(p-tolyl)boryl)oxy)ethanamine (7294-53-3)

To a suspension of magnesium (18.7 mmol, 455 mg) in a solution of diisopropylaminoborane (7.5 mmol, 1.2 mL) in anhydrous THF (30 mL), at room temperature, was added 855 mg of 4-bromotoluene. The mixture was then heated at 70 °C under stirring during 20 h. After cooling at room temperature, all the volatiles were removed under vacuum and to the resulting oil were added anhydrous THF (40 mL) and 1.40 g of 4-bromoanisole (7.5 mmol) and the mixture was heated at 70 °C during 20 h. After cooling at −78 °C, the reaction was quenched with a 3 M aqueous HCl solution (25 mL) and was stirred for 45 min at room temperature. To this solution was added water (20 mL), diethyl ether (20 mL), and the organic phase was separated. The aqueous phase was then extracted with DCM (6 × 30 mL) and the combined organic phases were dried over anhydrous Na_2_SO_4_, filtered and concentrated under reduced pressure. The residue was dissolved in Et_2_O (25 mL) and 1.2 eq of ethanolamine was added. Depending on substituents, precipitation occurs spontaneously or requires addition of pentane. Crystals were filtered, washed with pentane, and dried under vacuum to yield 2-(((4-methoxyphenyl)(p-tolyl)boryl)oxy)ethanamine as a white solid in 90% yield). ^1^H NMR (300 MHz, DMSO-*d*_6_): *δ* (ppm) 7.26 (m, 4H), 6.94 (d, *J* = 7.5 Hz, 2H), 6.71 (d, *J* = 8.4, 1.8 Hz, 2H), 5.89 (bt, 2H), 3.74 (t, *J* = 6.1 Hz, 2H), 3.67 (s, 3H), 2.80 (p, *J* = 6.1 Hz, 2H), 2.21 (s, 3H). ^11^B NMR (96 MHz, DMSO-*d*_6_): *δ* (ppm) 5.19 (s). ^13^C NMR (100 MHz, DMSO-*d*_6_): *δ* (ppm) 157.3, 133.3, 132.6, 131.6, 127.3, 112.3, 62.4, 54.6, 41.3, 20.9. Melting point: 154.3°C LC-MS (ESI): C_16_H_20_BNO_2_ 538.70 [2M+H]^+^: 539.32

### 4.9. Statistical Analysis

Given values are representative of at least three independent experiments and are given as mean ± SEM. When used, a *t*-test < 0.05 is considered as significant.

## 5. Conclusions

This study confirms that from the non-specific SOCE inhibitor, 2-APB, it is possible to create new analogues with more efficient properties. Thus, we designed, synthetized, and characterized 16 new molecules. Among them, the molecule we called P11 unveils interesting properties: Full SOCE inhibition for concentration less than 1 µM with an inhibition constant between 30 and 75 nM according to the cell lines, absence of effect on the two other main targets of 2-APB, SERCA and IP_3_R, absence of cytotoxicity on non-activated cells, but the ability to induce the caspase-3 activity and the apoptosis of PHA-activated cells. Thus, P11 is able to highjack the Ca^2+^ signaling of the T Jurkat cells from proliferation to death.

## 6. Patents

The 2-APB analogues are patented: FR1907184.

## Figures and Tables

**Table 1:** Inhibition constants of the various 2-APB analogs used in this study (left, compounds with a nanomolar *K*_i_, right, compounds with micromolar *K*_i_).

Compound	*K*_i_ (nM)	Compound	*K*_i_ (µM)
P11	75 ± 21	P13	1.8 ± 0.1
P6	275 ± 17	P5	2.1 ± 0.6
P15	294 ± 74	P16	3.1 ± 0.6
P8	350 ± 40	P3	3.5 ± 0.6
P12	374 ± 96	P2	3.5 ± 0.65
Dibenzothienyl-APB [7]	405 ± 23	P14	5.4 ± 0.3
P10	484 ± 116	P4	75 ± 21
P9	641 ± 103	P17	>> 100
P7	751 ± 97		

## Abbreviations

2-APB	2-aminoethyl diphenylborinate
[Ca^2+^]_cyt_	Cytosolic Ca^2+^ concentration
DAPI	di-aminodo Phenyl Indol
ELISA	Enzyme Linked Immunosorbent Assay
ER	Endoplasmic Reticulum
IL-2	Interleukin-2
IP_3_	Inositol 1,4,5-triphosphate
IP_3_R	IP_3_ receptor
K_i_	Inhibition constant
SOCC	Store-Operated Calcium Channel
SOCE	Store-Operated Calcium Entry
TG	Thapsigargin
TUNEL	Terminal Uridine Nick-End Labeling
SOCC	Store-Operated Calcium Channel

## References

[1] Berridge M.J., Lipp P., Bootman M.D. (2000). The versatility and universality of calcium signalling. Nat. Rev. Mol. Cell Biol..

[2] Parekh A.B., Putney J.W. (2005). Store-Operated Calcium Channels. Physiol. Rev..

[3] Feske S., Gwack Y., Prakriya M., Srikanth S., Puppel S.-H., Tanasa B., Hogan P.G., Lewis R.S., Daly M.J., Rao A. (2006). A mutation in Orai1 causes immune deficiency by abrogating CRAC channel function. Nat. Cell Biol..

[4] Putney J.W. (2010). Pharmacology of Store-operated Calcium Channels. Mol. Interv..

[5] Dobrydneva Y., Abelt C.J., Dovel B., Thadigiri C.M., Williams R.L., Blackmore P.F. (2005). 2-Aminoethoxydiphenyl Borate as a Prototype Drug for a Group of Structurally Related Calcium Channel Blockers in Human Platelets. Mol. Pharmacol..

[6] Goto J.-I., Suzuki A.Z., Ozaki S., Matsumoto N., Nakamura T., Ebisui E., Fleig A., Penner R., Mikoshiba K. (2010). Two novel 2-aminoethyl diphenylborinate (2-APB) analogues differentially activate and inhibit store-operated Ca2+ entry via STIM proteins. Cell Calcium..

[7] Djillani A., Nüße O., Dellis O. (2014). Characterization of novel store-operated calcium entry effectors. Biochim. et Biophys. Acta (BBA) - Bioenerg..

[8] Prakriya M., Lewis R.S. (2001). Potentiation and inhibition of Ca^2+^ release-activated Ca^2+^ channels by 2-aminoethyldiphenyl borate (2-APB) occurs independently of IP_3_ receptors. J. Physiol..

[9] Djillani A., Doignon I., Luyten T., Lamkhioued B., Gangloff S.C., Parys J.B., Nüße O., Chomienne C., Dellis O. (2015). Potentiation of the store-operated calcium entry (SOCE) induces phytohemagglutinin-activated Jurkat T cell apoptosis. Cell Calcium..

[10] Bittremieux M., Gerasimenko J.V., Schuermans M., Luyten T., Stapleton E., Alzayady K.J., De Smedt H., Yule D.I., Mikoshiba K., Vangheluwe P. (2017). DPB162-AE, an inhibitor of store-operated Ca2+ entry, can deplete the endoplasmic reticulum Ca2+ store. Cell Calcium..

[11] Schild A., Bhardwaj R., Wenger N., Tscherrig D., Kandasamy P., Dernič J., Baur R., Peinelt C., Hediger M.A., Lochner M. (2020). Synthesis and Pharmacological Characterization of 2-Aminoethyl Diphenylborinate (2-APB) Derivatives for Inhibition of Store-Operated Calcium Entry (SOCE) in MDA-MB-231 Breast Cancer Cells. Int. J. Mol. Sci..

[12] Ma H.-T., Venkatachalam K., Parys J.B., Gill D.L. (2001). Modification of Store-operated Channel Coupling and Inositol Trisphosphate Receptor Function by 2-Aminoethoxydiphenyl Borate in DT40 Lymphocytes. J. Biol. Chem..

[13] Bootman M.D., Collins T.J., Mackenzie L., Roderick H.L., Berridge M.J., Peppiatt C.M. (2002). 2-aminoethoxydiphenyl borate (2-APB) is a reliable blocker of store-operated Ca2+ entry but an inconsistent inhibitor of InsP3-induced Ca2+ release. FASEB J..

[14] Missiaen L., Callewaert G., De Smedt H., Parys J.B. (2001). 2-Aminoethoxydiphenyl borate affects the inositol 1,4,5-trisphosphate receptor, the intracellular Ca2+pump and the non-specific Ca2+leak from the non-mitochondrial Ca2+stores in permeabilized A7r5 cells. Cell Calcium..

[15] Peppiatt C.M., Collins T.J., MacKenzie L., Conway S.J., Holmes A.B., Bootman M.D., Berridge M.J., Seo J.T., Roderick H. (2003). 2-Aminoethoxydiphenyl borate (2-APB) antagonises inositol 1,4,5-trisphosphate-induced calcium release, inhibits calcium pumps and has a use-dependent and slowly reversible action on store-operated calcium entry channels. Cell Calcium..

[16] DeHaven W.I., Smyth J.T., Boyles R.R., Bird G.S., Putney J.W. (2008). Complex Actions of 2-Aminoethyldiphenyl Borate on Store-operated Calcium Entry. J. Biol. Chem..

[17] Peinelt C., Lis A., Beck A., Fleig A., Penner R. (2008). 2-Aminoethoxydiphenyl borate directly facilitates and indirectly inhibits STIM1-dependent gating of CRAC channels. J. Physiol..

[18] Zhang S.L., Kozak J.A., Jiang W., Yeromin A.V., Chen J., Yu Y., Penna A., Shen W., Chi V., Cahalan M.D. (2008). Store-dependent and -independent Modes Regulating Ca2+Release-activated Ca2+Channel Activity of Human Orai1 and Orai3. J. Biol. Chem..

[19] Rosenwasser L. (2010). Faculty Opinions recommendation of ORAI1 deficiency and lack of store-operated Ca2+ entry cause immunodeficiency, myopathy, and ectodermal dysplasia. Fac. Opin. Post Publ. Peer Rev. Biomed. Lit..

[20] Maruyama T., Kanaji T., Nakade S., Kanno T., Mikoshiba K. (1997). 2APB, 2-Aminoethoxydiphenyl Borate, a Membrane-Penetrable Modulator of Ins(1,4,5)P3-Induced Ca2+ Release. J. Biochem..

[21] Trebak M., Bird G.S., McKay R.R., Putney J.W. (2002). Comparison of human TRPC3 channels in receptor-activated and store-operated modes. Differential sensitivity to channel blockers suggests fundamental differences in channel composition. J Biol Chem..

[22] Chung S.C., McDonald T.V., Gardner P. (1994). Inhibition by SK&F 96365 of Ca2+ current, IL-2 production and activation in T lymphocytes. Br. J. Pharmacol..

[23] Tian C., Du L., Zhou Y., Li M. (2016). Store-operated CRAC channel inhibitors: Opportunities and challenges. Futur. Med. Chem..

[24] Putney J.W. (1986). A model for receptor-regulated calcium entry. Cell Calcium.

[25] Zhang S.L., Yu Y., Roos J., Kozak J.A., Deerinck T.J., Ellisman M.H., Stauderman K.A., Cahalan M.D. (2005). STIM1 is a Ca2+ sensor that activates CRAC channels and migrates from the Ca2+ store to the plasma membrane. Nat. Cell Biol..

[26] Feske S., Picard C., Fischer A. (2010). Immunodeficiency due to mutations in ORAI1 and STIM1. Clin. Immunol..

[27] Lacruz R.S., Feske S. (2015). Diseases caused by mutations inORAI1andSTIM1. Ann. New York Acad. Sci..

[28] Vaeth M., Yang J., Yamashita M., Zee I., Eckstein M., Knosp C., Kaufmann U., Jani P.K., Lacruz R.S., Flockerzi V. (2017). ORAI2 modulates store-operated calcium entry and T cell-mediated immunity. Nat. Commun..

[29] Motiani R.K., Abdullaev I.F., Trebak M. (2010). A novel native store-operated calcium channel encoded by Orai3:Selective requirement of Orai3 versus Orai1 in estrogen receptor positive versus estrogen receptor negative breast cancer cells. J. Biol. Chem..

[30] Prakriya M., Feske S., Gwack Y., Srikanth S., Rao A., Hogan P.G. (2006). Orai1 is an essential pore subunit of the CRAC channel. Nat. Cell Biol..

[31] Dickson E.J., Duman J.G., Moody M.W., Chen L., Hille B. (2012). Orai-STIM-mediated Ca2+ release from secretory granules revealed by a targeted Ca2+ and pH probe. Proc. Natl. Acad. Sci. USA.

[32] Varadarajan S., Tanaka K., Smalley J.L., Bampton E.T.W., Pellecchia M., Dinsdale D., Willars G.B., Cohen G.M. (2013). Endoplasmic Reticulum Membrane Reorganization Is Regulated by Ionic Homeostasis. PLoS ONE.

[33] Ikeya M., Yamanoue K., Mochizuki Y., Konishi H., Tadokoro S., Tanaka M., Suzuki R., Hirashima N. (2014). Orai-2 is localized on secretory granules and regulates antigen-evoked Ca2+ mobilization and exocytosis in mast cells. Biochem. Biophys. Res. Commun..

[34] Hou X., Pedi L., Diver M.M., Long S.B. (2012). Crystal structure of the calcium release-activated calcium channel Orai. Science.

[35] Zhou Y., Ramachandran S., Oh-Hora M., Rao A., Hogan P.G. (2010). Pore architecture of the ORAI1 store-operated calcium channel. Proc. Natl. Acad. Sci. USA.

[36] Yen M., Lokteva L.A., Lewis R.S. (2016). Functional Analysis of Orai1 Concatemers Supports a Hexameric Stoichiometry for the CRAC Channel. Biophys. J..

[37] Cai X., Zhou Y., Nwokonko R.M., Loktionova N.A., Wang X., Xin P., Trebak M., Wang Y., Gill D.L. (2016). The Orai1 Store-operated Calcium Channel Functions as a Hexamer. J. Biol. Chem..

[38] Dellis O., Mercier P., Chomienne C. (2011). The boron-oxygen core of borinate esters is responsible for the store-operated calcium entry potentiation ability. BMC Pharmacol..

[39] Scrimgeour N., Litjens T., Ma L., Barritt G.J., Rychkov G.Y. (2009). Properties of Orai1 mediated store-operated current depend on the expression levels of STIM1 and Orai1 proteins. J. Physiol..

[40] Liao Y., Erxleben C., Abramowitz J., Flockerzi V., Zhu M.X., Armstrong D.L., Birnbaumer L. (2008). Functional interactions among Orai1, TRPCs, and STIM1 suggest a STIM-regulated heteromeric Orai/TRPC model for SOCE/Icrac channels. Proc. Natl. Acad. Sci. USA.

[41] Zheng S., Zhou L., Ma G., Zhang T., Liu J., Li J., Nguyen N.T., Zhang X., Li W., Nwokonko R. (2018). Calcium store refilling and STIM activation in STIM- and Orai-deficient cell lines. Pflügers Archiv. Eur. J. Physiol..

[42] Miyawaki A., Furuichi T., Maeda N., Mikoshiba K. (1990). Expressed cerebellar-type inositol 1,4,5-trisphosphate receptor, P400, has calcium release activity in a fibroblast L cell line. Neuron.

[43] Grynkiewicz G., Poenie M., Tsien R.Y. (1985). A new generation of Ca2+ indicators with greatly improved fluorescence properties. J. Biol. Chem..

[44] Doignon I., Fayol O., Dellis O. (2019). Improvement of the rituximab–induced cell death by potentiation of the store-operated calcium entry in mantle cell lymphoma cell lines. Oncotarget.

[45] Dellis O., Gangloff S.C., Paulais M., Tondelier D., Rona J.-P., Brouillard F., Bouteau F., Guenounou M., Teulon J. (2002). Inhibition of the Calcium Release-activated Calcium (CRAC) Current in Jurkat T Cells by the HIV-1 Envelope Protein gp160. J. Biol. Chem..

[46] Brooks S., Storey K. (1992). Bound and determined: A computer program for making buffers of defined ion concentrations. Anal. Biochem..

[47] Luyten T., Bultynck G., Parys J.B., De Smedt H., Missiaen L. (2014). Measurement of intracellular Ca2+ release in permeabilized cells using 45Ca2+. Cold Spring Harb. Protoc..

[48] Parys J.B., De Smedt H. (2012). Inositol 1,4,5-Trisphosphate and Its Receptors. Adv. Exp. Med. Biol..

[49] Pinet S., Pucheault M., Richard J., Birepinte M., Charbonnier J.B., Liautard V. (2016). Borinic Acids via Direct Arylation of Amine–Borane Complexes: An Air- and Water-Stable Boron Source. Synthesis.

[50] Zhou H., Iwasaki H., Nakamura T., Nakamura K., Maruyama T., Hamano S.-I., Ozaki S., Mizutani A., Mikoshiba K. (2007). 2-Aminoethyl diphenylborinate analogues: Selective inhibition for store-operated Ca2+ entry. Biochem. Biophys. Res. Commun..

[51] Ohane K., Hayase Y. (2002). Nematicide for Plant Containing Organoboron Compound.

[52] Mikoshiba K., Iwasaki H., Maruyama T., Hamano S.-I. (2003). Intracellular Calcium Concentration Increase Inhibitors.

[53] Ozaki S., Suzuki A.Z., Bauer P.O., Ebisui E., Mikoshiba K. (2013). 2-Aminoethyl diphenylborinate (2-APB) analogues: Regulation of Ca2+ signaling. Biochem. Biophys. Res. Commun..

